# Unique Polymorphisms at *BCL11A*, *HBS1L-MYB* and *HBB* Loci Associated with HbF in Kuwaiti Patients with Sickle Cell Disease

**DOI:** 10.3390/jpm11060567

**Published:** 2021-06-17

**Authors:** Nagihan Akbulut-Jeradi, Maria Jinky Fernandez, Rasha Al Khaldi, Jalaja Sukumaran, Adekunle Adekile

**Affiliations:** 1 ATCLearn Center, Advanced Technology Company, P.O. Box 44558, Hawalli 32060, Kuwait; maria.f@atc.com.kw (M.J.F.); rasha.m@atc.com.kw (R.A.); 2 Department of Pediatrics, Faculty of Medicine, Kuwait University, P.O. Box 24923, Safat 13110, Kuwait; jalajasukumaran@hotmail.com (J.S.); adekunle.adekile@ku.edu.kw (A.A.)

**Keywords:** sickle cell disease, HbF modifiers, genetic association, single nucleotide polymorphism, Kuwait

## Abstract

Patients with sickle cell disease (SCD) in Kuwait have elevated HbF levels ranging from ~10–44%; however, the modulating factors are unclear. We investigated the association of single nucleotide polymorphisms (SNPs) at *BCL11A*, *HBS1L-MYB* and *HBB* with HbF levels in 237 Kuwaiti SCD patients, divided into 3 subgroups according to their HbF levels. Illumina Ampliseq custom DNA panel was used for genotyping and confirmed by arrayed primer extension or Sanger sequencing. In the *BCL11A* locus, the CC genotype of rs7606173 [χ^2^ = 16.5] and (GG) of rs10195871 [χ^2^ = 15.0] were associated with Hb-F1 and HbF-2 subgroups, unlike rs1427404-T [χ^2^ = 17.3], which showed the highest association across the three subgroups. *HBS1L-MYB* locus revealed 2 previously-described SNPs (rs66650371 [*χ*^2^ = 9.5] and rs35795442 [χ^2^ = 9.2]) and 2 previously-unreported SNPs, (rs13220662 [χ^2^ = 6.2] and rs1406811 [χ^2^ = 6.7]) that were associated with the HbF-3 subgroup, making this the key locus elevating HbF to the highest levels. *HBB* cluster variants were associated with lower levels of HbF (β = −1.1). We report four previously-unpublished variants showing significant association with HbF. Each of the three quantitative trait loci affects HbF levels differently; unique SNPs, especially in *HBS1L-MYB*, elevate HbF to the highest levels.

## 1. Introduction

Sickle cell disease (SCD) is the most widespread monogenic disease worldwide with significant morbidity and mortality, associated with the *HBB* mutation, rs334 (Glu6→Val; GAG→GTG). Homozygotes for this mutation have HbSS or sickle cell anemia (SCA), which is the most severe form of the disease. Compound heterozygotes such as HbSC or HbSβ-thal usually have less severe, but variable phenotypes. Irrespective of the Hb genotype, however, SCD shows remarkable clinical heterogeneity. Several genetic and environmental factors modulate the disease phenotype, of which the fetal hemoglobin (HbF) level is the most potent.

The eventual level of HbF (α_2_γ_2_) in patients with SCD is influenced by *cis*- and trans-acting single nucleotide polymorphisms (SNPs) in known quantitative trait loci (QTLs) on chromosomes 11 (*HBB*), 2 (*BCL11A*) and 6 (*HBS1L-MYB*), respectively. Several SNPs have been described in each of these loci in SCD and thalassemia patients in different parts of the world [1,2,3]. The important *cis*-acting SNPs on the *HBB* locus are also related to the βS gene cluster haplotype. Thus, patients with the Arab/Indian (AI) and Senegal (SEN) haplotypes have the highest HbF levels and mildest phenotypes. These are also the haplotypes associated with the *HBG2*, -158 (C→T) *XmnI* SNP, namely rs7482144.

Kuwait is a small country in the Northeast corner of the Arabian Peninsula; yet it has a very heterogeneous population, with the early settlers having migrated mainly from Eastern Saudi Arabia in the early 18th century, but included people from, Iran, Iraq, North and East Africa, the Mediterranean and Southern Asia. In spite of this, patients with SCD in Kuwait predominantly carry the AI haplotype, with elevated HbF levels, although there is considerable variability [4,5,6], with normally-distributed values ranging from ~10–40%. This suggests that there are multiple factors driving HbF expression in this group of patients. Previous investigations among patients in Kuwait and those from Eastern Saudi Arabia, carrying similar βS haplotypes, have failed to show a significant effect of modifier SNPs that were reported for patients from other populations [7,8,9]. It was therefore postulated that novel HbF-associated SNPs exist among Gulf Arabs, especially among patients with the highest HbF levels [8,10].

We have genotyped multiple SNPs at the *BCL11A*, *HBS1L-MYB*, *HBB* and Xp22 loci, to investigate whether unexplored SNPs are associated with high HbF levels in the Kuwaiti SCD population. The SNPs were selected based on detected association signals in a pilot study (unpublished data) and other common published variants. We categorized SCD patients according to their HbF levels into 3 subgroups (HbF-1, HbF-2 and HbF-3) and explored the association of the variants in the 3 QTLs with each subgroup. We hereby report novel variants at these loci, which have varying impacts on HbF expression across the 3 subgroups while some are unique to the group with the highest HbF levels. We take full cognizance of the need for large numbers of patients for this type of genomic study, therefore the data presented should be seen as preliminary, while further studies are underway.

## 2. Materials and Methods

The patients were drawn from consenting patients with SCD being followed in hematological clinics of Mubarak Al-Kabeer and Amiri Hospitals in Kuwait. The study was approved by the Human Research Ethics Committees of the Faculty of Medicine and the Kuwait Ministry of Health. The patients gave written consent or assent as appropriate.

Blood samples were drawn by venipuncture when the patients were in steady-state, i.e., without acute illness or crisis or having received blood transfusion in the 6 weeks preceding the study. Complete blood count (CBC) was obtained using an ABX Pentra 120 cell counter (ABX France, Montpellier), while Hb quantitation was achieved with cation-exchange high-performance liquid chromatography (HPLC) (Shimadzu LC-20AT, Shimadzu Corporation, Kyoto, Japan). Pre-treatment CBC and HbF values were used for data analysis among patients on hydroxyurea. The patients were divided into 3 subgroups based on their HbF levels: HbF-1 <20%, HbF-2 between 20 and 30% and HbF-3 with levels >30%.

### 2.1. Genotype Determination

Genomic DNA was extracted from peripheral leukocytes using the phenol-chloroform method. The Illumina Ampliseq custom DNA panel was used to genotype the DNA samples. For the *HBB* locus, all β-globin mutations and variants were confirmed by arrayed primer extension (APEX) or Sanger sequencing methods. The -158 (G→A) *XmnI* polymorphism in the *HBG2* gene promoter (rs7482144) was confirmed by digestion of an amplified fragment. Genomic variations of NGS data were analyzed with Alamut Visual v.2.11 and v.2.13 and Illumina’s Variant Interpreter software. APEX and Sanger sequencing results were analyzed using Genorama PicDb Autoscan 7.0 software (Asper Biotech) and GenomeLab GeXP Genetic Analysis System v. 11.0 (Beckman Coulter), respectively. β^S^ *HBB* haplotypes were determined using a modification of the phase SNP method described by Shaikho et al. [11]. Detailed review of the full haplotype descriptions and analysis results with the newly described significant SNPs as well as β-thalassemia mutations detected in this study will be reported separately.

### 2.2. Statistical Analysis

Descriptive analyses were performed with IBM SPSS software, version 25 (IBM, New York, NY, USA). The criterion for statistical significance was *p* < 0.05. HbF measurements achieved in patients younger than 5 years old were excluded from statistical analysis because HbF may not have stabilized before this age. All genetic analyses, quality control (QC) measures, SNPs association statistical tests and linkage disequilibrium calculations were performed using the PLINK software package version 1.9 (https://www.cog-genomics.org/plink2, accessed on 28 February 2020). First, SNPs with statistically significant (*p* < 0.05) deviation from the Hardy-Weinberg Equilibrium (HWE) were excluded from downstream analysis. Additionally, monomorphic (non-variable) SNPs or SNPs with a minor allele frequency (MAF) <5% (threshold) were excluded from further analysis. In order to identify SNPs that present non-redundant information about the genomic structure, tagging SNPs (tagSNPs) were selected on a block-by-block basis. The differences in HbF % and genotypes of the tentative SNPs were evaluated by one-way analysis of variance (ANOVA). The Haploview software package 4.2 (http://www.broad.mit.edu/haploview, accessed on 7 October 2019) was used to determine pairwise linkage disequilibrium across the genomic regions under study.

## 3. Results

### 3.1. General Characteristics

The study group consisted of 237 patients with SCD, made up of 65.8% HbSS and 34.2% HbSβ^0^ thalassemia. We included 112 healthy individuals in order to perform pre-statistical analysis among patients and healthy individuals and to generate haplotype patterns for the Kuwaiti population. Pre-statistical analysis revealed an overestimation of SNPs associated with the high HbF levels among the patients, thus the inclusion of healthy individuals failed to validate true association probably due to the wide significant differences (*p* ≤ 0.0001) in the HbF levels between patients (HbF mean = 23.3 ± 9.6%) and healthy individuals (HbF mean = 1.9 ± 2.3%) Therefore, the latter were excluded from genetic association analysis.

The mean age of the patients was 12.8 years with 44% females. There was no significant difference in the mean HbF values between the SS (22.7 ± 8.5%) and Sβ^0^-thal (24.1 ± 11.4%) groups. The mean HbF levels in the HbF-1, HbF-2 and HbF-3 subgroups were 16.8 ± 2.1, 23.4 ± 3.4, 34.3 ± 5.0%, respectively. The distribution of HbF levels among the patients is shown in Figure S1. Our results showed that HbF levels did not differ (*p* > 0.05) between the age groups. In the present analysis, HbF levels in female and male patients did not differ significantly (*p* > 0.05) in contrast to the previously published reports in the literature.

### 3.2. BCL11A Locus

**Figure 1:**
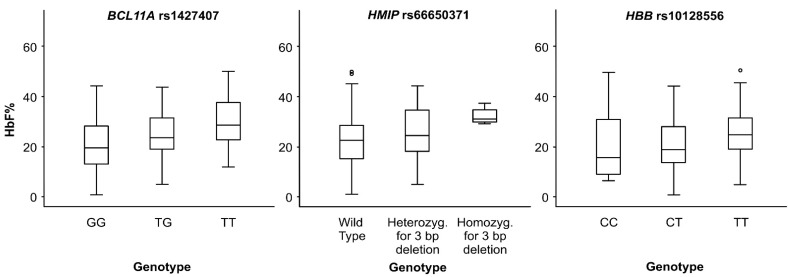
Box plots showing the relationship between HbF levels and the genotypes of rs1427407, rs66650371 and rs10128556. The values within the box range from 25th to 75th percentiles. The horizontal line within the box corresponds to the median value.

We genotyped 28 SNPs, 13 of which showed significant association in the three HbF subgroups. Association results for all variants are presented in Supplementary Material Table S1. Our major finding in this locus was an intronic variant, rs1427407, in the DNase I hypersensitive site (DHS) +62, which was in strong linkage disequilibrium (LD) (r^2^ ≥ 0.9) with three SNPs; rs766432, rs4671393, rs1896296; hence they are most likely tagging the same genetic signal. Subjects carrying the TT genotype of rs1427407 had the strongest association with HbF-2 subgroup (χ^2^ = 17.3, β = 1.7) (Table 1) and showed the highest HbF mean value as shown in Figure 1. The tagged SNPs showed similar significant trends in the HbF-2 subgroup (Table S1). The second significant association was rs7606173 (χ^2^ = 16.5 and *p* = 6.1 × 10^−5^) in DHS +55, which was in moderate LD (r^2^ = 0.5) with rs6709302. The third strongest was with rs10195871 (χ^2^ = 15 and *p* = 7.95 × 10^-5^), which was in strong LD (χ^2^ = 0.9) with rs10172646 and in moderate LD (r^2^ = 0.6) with rs11886868.

All five SNPs mentioned above showed relatively low significance in the HbF-3 subgroup. Subjects carrying homozygosity for the minor alleles rs7606173 (CC) and rs10195871 (GG) had relatively lower HbF levels compared to other genotypes. These findings were confirmed by the allelic change of the beta coefficient (β = −1.3 and β = −1.2, respectively) (Table 1).

Stepwise regression analysis revealed that two SNPs, rs1427407 and rs10195871, are independently associated with HbF levels. These two variants are located in *BCL11A* intron 2, and are in weak LD (0.38) with each other. To further understand the joint effect of the combinations of rs1427407, rs10195871 and rs7606173 on the *BCL11A* HbF association signal, we performed a haplotype analysis. The three SNPs generated five haplotypes that represent 98.9% of all haplotypes at this locus. TAG and GGC haplotypes were more strongly associated with HbF, explaining 11% and 10% of the phenotypic variation in HbF levels, respectively (Table 2). Thus, these haplotypes explain more phenotypic variance than the cumulative sum of rs1427407 in *BCL11A* taken individually (5.8%; Table 1). In this context, patients carrying the TAG haplotype had higher HbF levels (25.9%) compared to subjects carrying a GGC (15.3%) haplotype.

Interestingly, rs7569946, located in exon 4, was uniquely significant (χ^2^ = 7.5, *p* = 0.006) for the HbF-3 subgroup only. The considerable effect of the A allele at this locus (β = 0.59) results in an elevation of mean HbF levels for genotypes carrying the minor allele (Table 1).

### 3.3. HBS1L-MYB Intergenic Region

In this case, 40 SNPs were genotyped and 20 of them showed exceptional significance for HbF-3 subgroup (mean = 34.3 ± 5%) (Table S2). In this region, a large number of SNPs had similar allele frequencies and were in strong LD suggesting that these markers flag the same causal polymorphism. *HBS1L-MYB* polymorphisms *(HMIP)*, are distributed in three LD blocks [12]. The most effective one among these blocks is called *HMIP-2* that is divided into sub-loci *HMIP-2A* and *-2B*. *HMIP-2* was shown to influence disease severity in patients with SCD and beta thalassemia [13,14].

**Figure 2:**
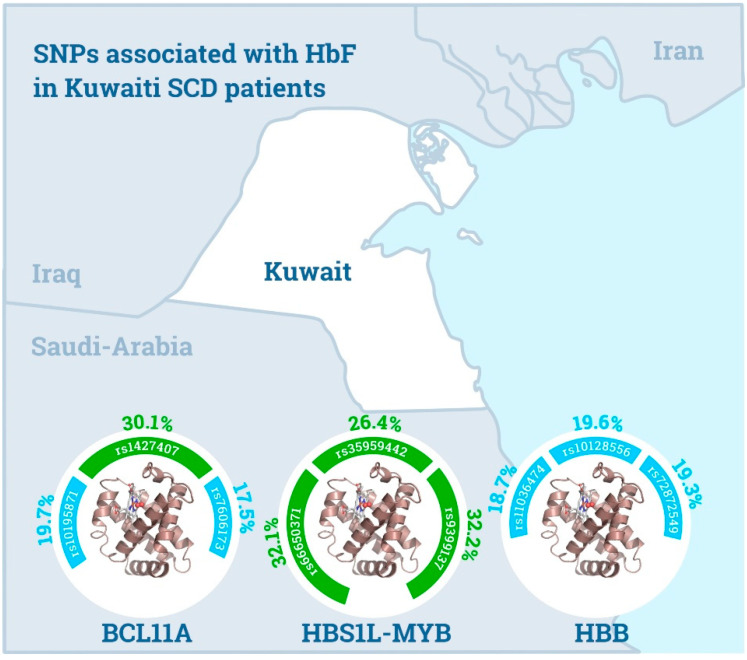
Schematic summary of variants affecting HbF levels in Kuwaiti SCD patients. The green arch represents the association with HbF-2 and HbF-3; the blue arch represents the association with HbF-1.

Our results highlighted 3 SNPs located in *HMIP-2A*; rs9399137, rs66650371 (3bp deletion) and rs35786788 that were in almost complete LD and conveyed the strongest impact in the HbF-3 subgroup (χ^2^~9.5 and *p* = 0.002), while in HbF-1 the impact of these 3 variants was diminished (*p* > 0.05) (Table 1). Figure 2 shows the relationship between HbF levels and rs66650371; the homozygous carrier of the 3-bp deletion had the highest HbF levels.

Another similar subgrouping trend was found for rs9494145, located in the *HMIP-2B* sub-locus; it was observed to be in strong LD with rs9483788 and in moderate LD with rs6920211. Subjects carrying the CC genotype of rs9494145 had the strongest association with the HbF-3 subgroup (χ^2^ = 8.5, *p* = 0.004). All of the three aforementioned SNPs maintained significant *p* values in HbF-2 and HbF-3 subgroups. In parallel, 3 other SNPs, rs4895441, rs9389269 and rs9402686 that were in strong LD, showed relatively less significant impact in the HbF-2 and HbF-3 subgroups (Table S2).

Notably, our findings revealed a different behavior of rs35959442 (previously named rs52090909), with a sole significance in the HbF-3 subgroup, similar to rs4895440 with which it was in strong LD. We also identified 4 other variants showing a similar trend in the HbF-3 subgroup (rs9402685, rs6930223, rs9376092 and rs9494142) but with less effect (Table S2).

Strikingly, we detected two previously-unpublished SNPs, rs13220662 and rs1406811, which were uniquely associated with HbF levels in the HbF-3 subgroup (Table 3). Indeed, the minor allele A of rs13220662 and rs1406811 had antagonistic effects on HbF levels. Similar to the previously-mentioned SNPs, rs34778774 also showed significance in the HbF-3 subgroup (Table S2).

We evaluated the effect of multiple variants in the *HBS1L-MYB* region to detect independent signals of association. Applying stepwise regression on 20 SNPs, we identified two SNPs, rs666750371 and rs35959442, which were independently associated with HbF levels with a weak LD (0.46) between the two. At the same time, the HbF phenotypic variation was 3.4% and 2.7% with rs666750371 and rs35959442, respectively (Table 1).

In order to investigate the effect of the other SNPs by excluding rs666750371 and rs35959442, we applied additional stepwise regression on 18 SNPs and found that rs34778774 and rs4895440 remained independently significant, with HbF phenotypic variations of 3% and 1.6%, respectively.

Haplotype analysis with the four aforementioned SNPs (rs666750371, rs35959442, rs34778774 and rs4895440) generated four haplotypes that represent 98% of all haplotypes at this locus. Subjects representing 22GT (2: TA, 2: CC, respectively) were associated with significantly higher HbF levels (mean = 26.4%). On the other hand, those with the 11CA (1: TACTA, 1: CCC, respectively) haplotype were associated with relatively lower HbF levels (18.7%). The variance explained by these two haplotypes was 3.4% and 3.8%, respectively (Table 2). Haplotypes identified in the *HBS1L-MYB* region did not show the trend of cumulative sum found in the haplotypes described in *BCL11A*.

### 3.4. HBB Locus

Genotyping was performed for 58 SNPs across the β-globin region that includes the locus control region (LCR) and the *HBE1*, *HBG2*, *HBG1*, *HBBP1*, *HBD* and *HBB* genes. Analysis of the markers showed a pattern of high LD across the entire *HBB* region; rs7482144 was in LD (≤0.7) with rs2855122, rs2855121, rs4910740, rs72872549 and rs67385638 but did not show the strongest impact on HbF levels. The latter SNP, rs67385638, which is an intronic variant of the *HBE1* gene, showed the strongest association in HbF-1 and HbF-2 subgroups (χ^2^ = 19.2 and χ^2^ = 19.8, *p* = 1.7 × 10^−5^ and *p* = 10 × 10^−6^, respectively) (Table 1). Other SNPs showing a stronger association than rs7482144 were rs11036474 (*HBG2*) and rs10128556 located downstream of *HBG1* (Table 1). In that context, subjects carrying the CC genotype of rs10128556 had the lowest HbF levels (Figure 1). SNPs rs11036474, rs2855039 and rs10128556, rs2071348 tagged the same signal (Table 1 and Table S3).

Conditional analysis on rs11036474 and rs10128556 caused rs7482144 to lose its significant association with HbF. However, rs7482144 maintained its significance when it was conditioned on rs67385638. These findings clearly indicate that rs7482144 is not the only variant that causes the robust effect on HbF levels (mean = 16.7±2.1%) in Kuwaiti patients with SCD.

A previously unpublished intronic variant of the *HBE1* gene, rs72872549, showed a similar trend but with a more significant association than rs7482144, which were both in moderate LD (χ^2^ = 16.5 vs. χ^2^ = 15.2, *p* = 4.95 × 10^−5^ vs. *p* = 2.00 × 10^−4^, respectively; Table 1). We identified two more SNPs, rs7937649 *(OR51V1)* and rs3813726 *(HBD)*, which were both in moderate LD and found to be significantly associated with HbF (χ^2^ = 13.5, *p* = 0.0003, χ^2^ = 10.2, *p* = 0.001, respectively). It is noteworthy that rs3813726 has not been reported before (Table 3). There was a significant independent association of rs416586 *(OR51B5)* with HbF (χ^2^ = 13.3, *p* = 0.005) and it was not in LD with any other SNP (Table S3).

Almost all the SNPs in the *HBB* locus in this study showed a significant association with HbF levels in the lower (HbF-1) and middle (HbF-2) subgroups. Therefore, identified minor alleles of studied variants in this locus had a negative impact on HbF levels; this was confirmed by the allelic change of beta coefficients. Notably, rs3759071 showed significance solely for the highest subgroup (HbF-3), as confirmed by the allelic change (G) of the beta coefficient (Table 1).

We performed haplotype analysis with the five variants rs10128556, rs11036474, rs7482144, rs72872549 and rs67385638, including a newly-identified SNP, and generated three haplotypes that represent 97.2% of all haplotypes in this locus. 72% of the SCD subjects carrying the TCATG haplotype had higher levels of HbF (mean = 24%); on the other hand, the CTGCC haplotype was associated with lower HbF (mean = 16.2%) levels. Haplotypes identified in this locus explained the phenotypic variation in HbF by 3.1% and 6%, respectively (Table 2).

Figure 2 depicts the main findings of our study, showing the selected variants in QTLs (*BCL11A*, *HBS1L-MYB* and *HBB*) which are strongly associated with HbF levels. Each SNP is displayed with its regulatory effect (green arch: associated with HbF-2 and HbF-3; blue arch: associated with HbF-1) with mean HbF % values.

### 3.5. Chromosome X Associations

Four SNPs on chromosome X were studied; a moderately strong association with HbF levels was found for rs4969549 in Xp22.11, and for rs12559632 in Xp22.2 (PHEX). Rs12559632 showed significant results in the HbF-2 and HbF-3 groups (χ^2^ = 4.9 and χ^2^ = 7.9, *p* = 0.026 and *p* = 0.005, respectively) while rs4969549 was significant only in HbF-3 (χ^2^ = 9.6 and *p* = 0.008) (Table S4).

## 4. Discussion

The Gaussian distribution of HbF levels among patients with the AI haplotype gives a unique opportunity to investigate the genomic drivers of HbF expression in SCD. Even though Kuwaiti patients generally have elevated HbF levels, there is still a marked variability, leading us to hypothesize that a variety of genetic modifiers act in a stepwise and probably synergistic manner, to drive HbF expression in this group. In order to investigate the variants associated with different degrees of HbF elevation among our patients, we divided the patients into subgroups according to their HbF levels. 

Previous genomic studies have identified several SNPs from QTLs on chromosomes 2p15, 6q23 and 11p16 in association with HbF levels and /F-cell numbers [1,3,13,15]. Many of the reported SNPs likely tag the same genetic signal at each locus since they show moderate to high LD. Indeed, our study confirms that polymorphisms in the *BCL11A*, *MYB-HBS1L*, as well as the *HBB* locus, are associated with HbF levels. 

*BCL11A* is a major regulator of hemoglobin gene switching [16] and a direct repressor of HbF production [17]. Polymorphisms within the 14 kb intron 2 of *BCL11A* are associated with HbF levels in different populations [3,13,18]. In the present study, *BCL11A* is the most influential HbF modifier locus, affecting each of the HbF subgroups. We found that rs1427407 in DHS +62 had the strongest association with HbF, especially in the HbF-2 subgroup. The G→T change alters the DNA sequence for a key regulatory element ‘+62’ within the erythroid intronic enhancer for *BCL11A* [19]. This variant could be causal to HbF association seen with other closely-linked markers in the locus, such as rs766432, rs4671393 and rs1896296. Another SNP, namely rs10195871, was reported to be associated with HbF in several populations [18,20,21], and our study is the first to report this association among Gulf Arabs. Our results clearly indicate that rs10195871 and rs10172646, which were in strong LD, are significant for every HbF subgroup, with the highest significance in HbF-1. Consistent with previous reports [9,18,22], we also found that rs7606173 in DHS +55, is the genetic marker that was strongly associated with HbF, especially in the HbF-1 subgroup.

Our results not only confirm the previously-associated *BCL11A* SNPs, but also reveal an independent effect of rs1427407 and rs10195871 on HbF regulation. Haplotype analysis showed that this group of SNPs has a stronger impact than individual ones, consistent with the hypothesis [19] that multiple functional SNPs within the composite enhancer act synergistically to affect *BCL11A* regulation. HbF phenotypic variation of the synonymous variant, rs7569946, within exon-4 in *BCL11A*, was limited to 1.8%, indicating its unique specificity and significance to the highest HbF levels (HbF-3). Further investigation of this variant is needed to confirm its effect in SCD patients with non-AI β^S^ haplotypes and low HbF levels.

Polymorphisms in the *HBS1L-MYB* locus are strongly associated with HbF levels among European and Chinese patients with thalassemia and SCD, but are not so significant among African [13,23] and Saudi patients [8]. However, our results showed that SNPs rs66650371, rs9399137 and rs35786788, in this QTL are significantly associated with the “super-HbF expressors” in subgroup HbF-3. The 3-bp deletion rs66650371, located near the erythroid-specific DNase I hypersensitive site 2 within block 2, has previously been shown to be strongly associated with HbF [23]. This site is surrounded by binding sites for erythroid-specific transcription factors such as TAL1/E47, GATA, RUNX1, LDB1 and KLF1, and was proposed to be a major factor contributing to elevate HbF to high levels [23,24,25]. In this regard, the 3-bp deletion is likely of direct functional significance for critical regulatory elements within the core enhancer for MYB, which encodes an important erythroid transcription factor [23,24].

Patients carrying rs66650371 had uniquely high HbF levels, thus making it probably the most functional and independent variant within *HBS1L-MYB* to elevate HbF to the highest levels. Similar results were reported for rs9399137, which was in complete LD with rs66650371 in African American and Tanzanian patients with sickle cell anemia [26,27]. The phenotypic variance explained by the defined haplotypes did not show higher magnitude than individual SNP effects, indicating that those variants within *HMIP* act independently to elevate HbF to the extraordinary levels in the Kuwaiti population.

The *HBB* locus has been extensively studied, and several genetic modifiers of HbF have been detected within the cluster. The *XmnI* polymorphism, rs7482144, in the proximal promoter of *HBG2*, tags the AI and SEN haplotypes; its association with HbF levels is well established in different populations [13,16]. The results of our study confirm this association; in addition, we found other variants within this cluster that have an equal or even stronger effect on HbF levels. For example, rs67385638, rs11036474 and rs10128556 were more strongly associated with HbF levels than rs7482144. Conditional analysis showed that rs11036474 and rs10128556 were the most independently-associated variants found in the HbF-1 and HbF-2 subgroups while rs11036474 has not been reported in other populations except in China [28]; our findings on rs10128556 corroborate those of Galarneu et al. [6].

The haplotypes generated by rs10138556, rs11036474, rs7482144, rs72872549 and rs67385638 in the *HBB* cluster confirmed the negative impact of minor alleles. Indeed, subjects carrying the CTGCC haplotype had 8% lower HbF levels than the ones carrying TCATG. The identified haplotypes explained the phenotypic variation in HbF by 3.1% and 6%, respectively. The observed variances may not reflect the real effect of all synergistic SNPs, which are in strong LD and acting together in a poly-variant manner in the HBB locus. Individual minor alleles of rs10128556-C, rs11036474-T, rs7482144-G, rs72872549-C and rs67385638-C had a relatively lower negative effect than CTGCC haplotype. Additionally, our findings indicated that the group effect of the major alleles of the five variants used for haplotype analysis might have a higher effect of HbF levels tentatively. The effects of the variants reported in this study indicated that SNPs in the *HBB* locus were specific to the HbF-1 subgroup. This phenomenon was confirmed by the allelic change of beta coefficients.

In chromosome X, we found two SNPs (rs4969549 and rs12559632) that were also reported by other studies [15,18]. However, the significance of these variants did not surpass a *p* value of 0.005 which suggests that the X-linked factor influencing HbF production is not crucial in patients with the AI haplotype [29].

The independent as well as the synergistic effects of variants in the *BCL11A* locus show that this region is associated with HbF across the 3 subgroups, while some variants especially in the *HBS1L-MYB* locus are independent key players, elevating HbF to the highest observed levels. Thus, these “super HbF expressors” probably represent a unique group; the identification of the genetic variants associated with them may provide new therapeutic options for SCD. Since most patients with SCD in Kuwait carry the AI haplotype, the assumption was that their high HbF levels were primarily attributable to rs7482144. However, the results of the present study and others have shown that, even within the *HBB*, there are other SNPs that are more potent. Validation of our hypothesis requires the study of a large number of patients, which unfortunately are not available in Kuwait. For this reason, collaborative studies involving other countries in the region are underway. The present results are, therefore preliminary, pilot data.

## Figures and Tables

**Table 1:** Variants in *BCL11A*, *HBS1L-MYB* and *HBB* loci showing the most significant association with HbF in Kuwaiti patients with SCD.

Locus	SNP ID	BP	MAF	A1	% R^2^	HbF-1		HbF-2		HbF-3		β	*p*
						χ^2^	*p*	χ^2^	*p*	χ^2^	*p*		
*BCL11A*	rs1427407	60490908	0.2975	T	5.80	14.86	0.0003	17.31	3.32 × 10^−5^	9.97	0.0087	1.65	0.0023
	rs7606173	60498316	0.3249	C	1.70	16.46	6.13 × 10^−5^	9.99	0.0143	8.53	0.0103	−1.27	9.17 × 10^−5^
	rs10195871	60493454	0.4916	G	3.40	15.00	7.95 × 10^−5^	10.66	0.0234	6.22	0.0126	−1.21	0.0003
	rs7569946	60460824	0.3207	A	1.80	3.21	0.13169	0.80	0.4420	7.51	0.0061	0.59	0.0073
*HBS1L-MYB*	rs9399137	135097880	0.1181	C	0.10	3.33	0.1090	4.96	0.0259	9.49	0.0021	0.78	0.0056
	rs66650371	135097495	0.1181	2 *	3.40	3.33	0.1090	5.33	0.0214	9.49	0.0021	0.78	0.0056
	rs35786788	135097904	0.1181	A	0.20	3.33	0.1090	4.96	0.0259	9.17	0.0015	0.76	0.0043
	rs35959442	135103041	0.1983	G	2.70	1.60	0.3053	2.77	0.0960	9.19	0.0025	0.76	0.0026
*HBB*	rs67385638	5269140	0.2089	C	1.70	19.22	1.65 × 10^−5^	19.82	9.96 × 10^−6^	3.65	0.1641	−1.13	0.0002
	rs11036474	1253948	0.2131	T	1.50	18.13	2.65 × 10^−5^	18.22	2.44 × 10^−5^	3.43	0.0963	−1.08	0.0002
	rs10128556	5242453	0.2152	C	1.30	17.61	3.34 × 10^−5^	17.47	3.76 × 10^−5^	3.11	0.1849	−1.07	0.0003
	rs72872549	5268823	0.2152	C	1.70	16.48	4.95 × 10^−5^	15.45	0.0002	3.11	0.1849	−1.07	0.0003
	rs7482144	5254939	0.2574	G	1.20	15.25	0.0002	16.04	9.02 × 10^−5^	2.59	0.3364	−1.08	0.0005
	rs3759071	5270302	0.1160	G	0.20	1.45	0.2699	3.72	0.0804	8.55	0.0044	0.70	0.0100

* rs66650371 genotypes codes are (1: TACTA, 2: TA), A1 indicates Minor Allele; β, Beta Coefficient; *BCL11A*, B-cell lymphoma/leukemia 11A; BP, Physical Position; *HBB*, beta globin; *HBS1L-MYB*, intergenic region between GTP-binding elongation factor *HBS1L* and myeloblastosis oncogene MYB; ID, Identification; MAF, Minor Allele Frequency; % R^2^, Variance Explained; χ^2^, Chi-square.

**Table 2:** Haplotype analysis of selected SNPs located in the 3 principal loci *BCL11A, HBS1L-MYB* and *HBB* that are associated with HbF levels in single marker analysis of Kuwaiti patients with SCD.

Locus	Haplotype	HbF %	β	% R^2^	% Frequency	*p*	SNPs
*BCL11A*	TAG	25.96	4.83	11.00	27.91	3.00 × 10^−7^	rs1427407|rs10195871|rs7606173
	GGC	15.30	−4.94	10.00	30.12	9.59 × 10^−7^	rs1427407|rs10195871|rs7606173
*HBS1L-MYB*	22GT *	26.35	3.80	3.40	11.14	0.0040	rs66650371|rs34778774|rs35959442|rs4895440
	11CA **	18.66	−3.13	3.80	77.02	0.0030	rs66650371|rs34778774|rs35959442|rs4895440
*HBB*	TCATG	24.00	2.49	3.10	72.14	0.0010	rs10128556|rs11036474|rs7482144|rs72872549|rs67385638
	CTGCC	16.20	−3.40	6.00	19.36	0.0110	rs10128556|rs11036474|rs7482144|rs72872549|rs67385638

* rs66650371 genotypes codes are (1: TACTA, 2: TA); ** rs34778774 genotypes codes are (1: CCC, 2: CC), β, Beta Coefficient; *BCL11A*, B-cell lymphoma/leukemia 11A; *HBB*, beta globin; *HBS1L-MYB*, intergenic region between GTP-binding elongation factor *HBS1L* and myeloblastosis oncogene MYB; % R^2^, Variance Explained.

**Table 3:** Previously Unpublished Variants in *HBS1L-MYB*, and *HBB* loci showing significant association with HbF in Kuwaiti patients with SCD.

Locus	SNP ID	BP	MAF	A1	% R^2^	HbF-1		HbF-2		HbF-3		β	*p*
						χ^2^	*p*	χ^2^	*p*	χ^2^	*p*		
*HBS1L-MYB*	rs13220662	135074410	0.3418	A	1.5	1.66	0.2857	2.82	0.0930	6.16	0.0303	0.45	0.0387
	rs1406811	135118989	0.4726	A	1.6	0.31	0.5904	0.18	0.6737	6.72	0.0096	−0.51	0.0118
*HBB*	rs3813726	5234759	0.2574	T	0.8	10.20	0.0014	8.81	0.0041	0.95	0.5765	−0.83	0.0033
	rs72872549	5268823	0.2152	C	1.7	16.48	4.95 × 10^−5^	15.45	0.0001	3.11	0.1849	−1.07	0.0003

A1 indicates Minor Allele; β, Beta Coefficient; *BCL11A*, B-cell lymphoma/leukemia 11A; BP, Physical Position; *HBB*, beta globin; *HBS1L-MYB*, intergenic region between GTP-binding elongation factor *HBS1L* and myeloblastosis oncogene MYB; ID, Identification; MAF, Minor Allele Frequency; % R^2^, Variance Explained; χ^2^, Chi-square.

## Data Availability

More of the relevant data from the study are provided as supplementary material to this paper, while all data are available on demand from the first author.

## Supplementary Materials

The following are available online at https://www.mdpi.com/article/10.3390/jpm11060567/s1, Figure S1: Distribution of HbF levels among patients, Table S1: Fetal hemoglobin association results for 24 SNPs at the *BCL11A* locus in Kuwaiti patients with SCD, Table S2: Fetal hemoglobin association results for 36 SNPs at the *HBS1L-MYB* locus in Kuwaiti patients with SCD, Table S3: Fetal hemoglobin association results for 52 SNPs at the *HBB* locus in Kuwaiti patients with SCD, Table S4: Fetal hemoglobin association results for 4 SNPs at the ChrX in Kuwaiti patients with SCD.

## References

[1] Menzel S., Jiang J., Silver N., Gallagher J., Cunningham J., Surdulescu G., Lathrop M., Farrall M., Spector T.D., Thein S.L. (2007). The HBS1L-MYB intergenic region on chromosome 6q23.3 influences erythrocyte, platelet, and monocyte counts in humans. Blood.

[2] Thein S.L., Menzel S. (2009). Discovering the genetics underlying foetal haemoglobin production in adults. Br. J. Haematol..

[3] Uda M., Galanello R., Sanna S., Lettre G., Sankaran V.G., Chen W., Usala G., Busonero F., Maschio A., Albai G. (2008). Genome-wide association study shows BCL11A associated with persistent fetal hemoglobin and amelioration of the phenotype of beta-thalassemia. Proc. Natl. Acad. Sci. USA.

[4] Adekile A.D., Gu L.H., Baysal E., Haider M.Z., Al-Fuzae L., Aboobacker K.C., Al-Rashied A., Huisman T.H. (1994). Molecular characterization of alpha-thalassemia determinants, beta-thalassemia alleles, and beta S haplotypes among Kuwaiti Arabs. Acta Haematol..

[5] Adekile A.D., Haider M.Z. (1996). Morbidity, beta S haplotype and alpha-globin gene patterns among sickle cell anemia patients in Kuwait. Acta Haematol..

[6] Galarneau G., Palmer C.D., Sankaran V.G., Orkin S.H., Hirschhorn J.N., Lettre G. (2010). Fine-mapping at three loci known to affect fetal hemoglobin levels explains additional genetic variation. Nat. Genet..

[7] Adekile A., Menzel S., Gupta R., Al-Sharida S., Farag A., Haider M., Akbulut N., Mustafa N., Thein S.L. (2015). Response to hydroxyurea among kuwaiti patients with sickle cell disease and elevated baseline HbF levels. Am. J. Hematol..

[8] Ngo D., Bae H., Steinberg M.H., Sebastiani P., Solovieff N., Baldwin C.T., Melista E., Safaya S., Farrer L.A., Al-Suliman A.M. (2013). Fetal hemoglobin in sickle cell anemia: Genetic studies of the Arab-Indian haplotype. Blood Cells Mol. Dis..

[9] Shaikho E.M., Farrell J.J., Alsultan A., Sebastiani P., Steinberg M.H. (2017). Genetic determinants of HbF in Saudi Arabian and African Benin haplotype sickle cell anemia. Am. J. Hematol..

[10] Adekile A. (2020). The Genetic and Clinical Significance of Fetal Hemoglobin Expression in Sickle Cell Disease. Med. Princ. Pr..

[11] Shaikho E.M., Farrell J.J., Alsultan A., Qutub H., Al-Ali A.K., Figueiredo M.S., Chui D.H., Farrer L., Murphy G.J., Mostoslavsky G. (2017). A phased SNP-based classification of sickle cell anemia HBB haplotypes. BMC Genom..

[12] Thein S.L., Menzel S., Peng X., Best S., Jiang J., Close J., Silver N., Gerovasilli A., Ping C., Yamaguchi M. (2007). Intergenic variants of HBS1L-MYB are responsible for a major quantitative trait locus on chromosome 6q23 influencing fetal hemoglobin levels in adults. Proc. Natl. Acad. Sci. USA.

[13] Lettre G., Sankaran V.G., Bezerra M.A., Araújo A.S., Uda M., Sanna S., Cao A., Schlessinger D., Costa F.F., Hirschhorn J.N. (2008). DNA polymorphisms at the BCL11A, HBS1L-MYB, and beta-globin loci associate with fetal hemoglobin levels and pain crises in sickle cell disease. Proc. Natl. Acad. Sci. USA.

[14] Galanello R., Sanna S., Perseu L., Sollaino M.C., Satta S., Lai M.E., Barella S., Uda M., Usala G., Abecasis G.R. (2009). Ame-lioration of Sardinian beta0 thalassemia by genetic modifiers. Blood.

[15] Solovieff N., Milton J.N., Hartley S.W., Sherva R., Sebastiani P., Dworkis D.A., Klings E.S., Farrer L.A., Garrett M.E., Ashley-Koch A. (2010). Fetal hemoglobin in sickle cell anemia: Genome-wide association studies suggest a regulatory region in the 5′ olfactory receptor gene cluster. Blood.

[16] Menzel S., Garner C., Gut I., Matsuda F., Yamaguchi M., Heath S., Foglio M., Zelenika D., Boland A., Rooks H. (2007). A QTL influencing F cell production maps to a gene encoding a zinc-finger protein on chro-mosome 2p15. Nat. Genet..

[17] Sankaran V.G., Menne T.F., Xu J., Akie T.E., Lettre G., Van Handel B., Mikkola H.K.A., Hirschhorn J.N., Cantor A.B., Orkin S.H. (2008). Human Fetal Hemoglobin Expression Is Regulated by the Developmental Stage-Specific Repressor BCL11A. Science.

[18] Bhatnagar P., Purvis S., Barron-Casella E., DeBaun M.R., Casella J.F., Arking D.E., Keefer J.R. (2011). Genome-wide association study identifies genetic variants influencing F-cell levels in sickle-cell patients. J. Hum. Genet..

[19] Bauer D.E., Kamran S.C., Lessard S., Xu J., Fujiwara Y., Lin C., Shao Z., Canver M.C., Smith E.C., Pinello L. (2013). An Erythroid Enhancer of BCL11A Subject to Genetic Variation Determines Fetal Hemoglobin Level. Science.

[20] Griffin P.J., Sebastiani P., Edward H., Baldwin C.T., Gladwin M.T., Gordeuk V.R., Chui D., Steinberg M.H. (2014). The genetics of hemoglobin A2regulation in sickle cell anemia. Am. J. Hematol..

[21] Liu L., Pertsemlidis A., Ding L.-H., Story M.D., Steinberg M.H., Sebastiani P., Hoppe C., Ballas S.K., Pace B.S. (2016). Original Research: A case-control genome-wide association study identifies genetic modifiers of fetal hemoglobin in sickle cell disease. Exp. Biol. Med..

[22] Sebastiani P., Farrell J., Alsultan A., Wang S., Edward H.L., Shappell H., Bae H., Milton J.N., Baldwin C., Al-Rubaish A. (2015). BCL11A enhancer haplotypes and fetal hemoglobin in sickle cell anemia. Blood Cells Mol. Dis..

[23] Farrell J.J., Sherva R.M., Chen Z.-Y., Luo H.-Y., Chu B.F., Ha S.Y., Li C.K., Lee A.C.W., Li R.C.H., Yuen H.L. (2011). A 3-bp deletion in the HBS1L-MYB intergenic region on chromosome 6q23 is associated with HbF expression. Blood.

[24] Stadhouders R., Aktuna S., Thongjuea S., Aghajanirefah A., Pourfarzad F., van Ijcken W., Lenhard B., Rooks H., Best S., Menzel S. (2014). HBS1L-MYB intergenic variants modulate fetal hemoglobin via long-range MYB enhancers. J. Clin. Investig..

[25] Canver M.C., Lessard S., Pinello L., Wu Y., Ilboudo Y., Stern E.N., Needleman A.J., Galactéros F., Brugnara C., Kutlar A. (2017). Variant-aware saturating mutagenesis using multiple Cas9 nucleases identifies regulatory elements at trait-associated loci. Nat. Genet..

[26] Akinsheye I., Solovieff N., Ngo D., Malek A., Sebastiani P., Steinberg M.H., Chui D.H.l. (2012). Fetal hemoglobin in sickle cell anemia: Molecular characterization of the unusually high fetal hemoglobin phenotype in African Americans. Am. J. Hematol..

[27] Adeyemo T.A., Ojewunmi O.O., Oyetunji I.A., Rooks H., Rees D.C., Akinsulie A.O., Akanmu A.S., Thein S.L., Menzel S. (2018). A survey of genetic fetal-haemoglobin modifiers in Nigerian patients with sickle cell anaemia. PLoS ONE.

[28] Zhang L., Zhang Q., Tang Y., Cong P., Ye Y., Chen S., Zhang X., Chen Y., Zhu B., Cai W. (2019). LOVD–DASH: A comprehensive LOVD database coupled with diagnosis and an at-risk assessment system for hemoglobinopathies. Hum. Mutat..

[29] Adekile A., Al-Kandari M., Haider M., Rajaa M., D’Souza M., Sukumaran J. (2007). Hemoglobin F Concentration as a Function of Age in Kuwaiti Sickle Cell Disease Patients. Med Princ. Pr..

