# Supplementary material for: Unique Polymorphisms at BCL11A, HBS1L-MYB and HBB Loci Associated with HbF in Kuwaiti Patients with Sickle Cell Disease
Source: J Pers Med. 2021 Jun 17;11(6):567. doi: 10.3390/jpm11060567 (PMC8234980; doi:10.3390/jpm11060567)
Supplement: Supplementary file 1 [file jpm-11-00567-s001.zip › jpm-1228873-supplementary.pdf]

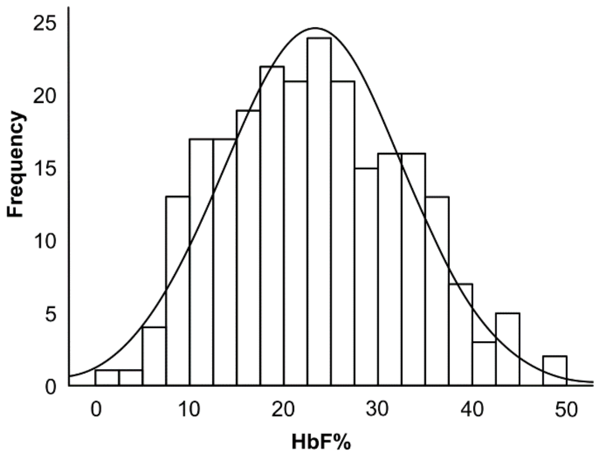

Figure S1. Distribution of HbF levels among patients.

Table S1. Fetal hemoglobin association results for 24 SNPs at the BCL11A locus in Kuwaiti patients with SCD

Locus	SNP ID	BP	MAF	A1	HbF-1		HbF-2		HbF-3		β	*P*
					χ^2	*P*	χ^2	*P*	χ^2	*P*		
BCL11A	rs766432	60492835	0.2975	C	10.67	0.0035	15.00	0.0001	9.09	0.0100	1.16	0.0079
	rs4671393	60493816	0.2932	A	11.11	0.0040	15.37	8.9×10^{-5}	9.77	0.0149	1.18	0.0084
	rs1896296	60496952	0.2932	G	11.11	0.0040	15.37	8.9×10^{-5}	9.38	0.0163	1.20	0.0079
	rs6709302	60500494	0.2553	A	9.20	0.0075	9.02	0.0091	4.15	0.0532	-0.85	0.0090
	rs10172646	60493622	0.4958	A	14.53	0.0001	9.09	0.0330	5.98	0.0161	-1.14	0.0004
	rs11886868	60493111	0.3136	T	13.50	0.0001	7.97	0.0050	7.48	0.0060	-0.83	0.0005
	rs74946846	60481423	0.0042	A	0.38	0.5613	0.33	0.6058	0.33	0.6228	ND	ND
	rs6732518	60481462	0.4873	T	13.61	0.0003	8.55	0.0200	6.25	0.0183	-1.07	0.0007
	rs10189857	60486100	0.3987	G	0.63	0.5260	0.71	0.5113	0.56	0.5063	0.23	0.5285
	rs6545816	60487726	0.4810	A	3.81	0.0558	4.28	0.0488	2.06	0.2160	0.56	0.0633
	rs7599488	60491212	0.3966	T	1.10	0.4023	0.65	0.47115	0.39	0.5775	0.31	0.4070
	rs1896294	60491939	0.4937	T	14.82	0.0001	9.42	0.0279	5.27	0.0277	-1.14	0.0004
	rs766431	60492783	0.0781	G	2.65	0.1200	0.93	0.4959	0.84	0.5495	ND	ND
	rs944022401	60493764	0.0021	G	0.19	0.6794	0.47	0.5041	2.13	0.1629	ND	ND
	rs571634152	60494172	0	0	ND	ND	ND	ND	ND	ND	ND	ND
	rs7584113	60494176	0.4937	A	13.11	0.0003	7.56	0.0300	6.76	0.0132	1.10	0.0007
	rs7557939	60494212	0.5000	G	13.95	0.0002	8.45	0.0413	6.63	0.0109	1.10	0.0006
	rs6706648	60494905	0.1878	T	4.07	0.1125	5.01	0.0372	1.66	0.2045	-0.60	0.1105
	rs6738440	60495106	0.1245	G	2.56	0.1487	3.64	0.0565	2.53	0.1894	-0.52	0.1625
	rs7565301	60496131	0.1793	A	0.36	0.5979	0.45	0.5678	0.12	0.8012	0.23	0.6059
	rs6729815	60496537	0.4831	T	2.03	0.1554	3.01	0.1063	1.88	0.2372	0.39	0.1676
	rs186824634	60496588	0.0042	A	15.01	0.0041	5.01	0.0444	1.06	0.3165	-9.04	0.9928
	rs1896295	60496951	0.2764	T	8.39	0.0075	10.99	0.0009	2.93	0.0907	1.06	0.0132
	rs567628535	60500449	0.0084	A	0.18	0.7260	0.19	0.7230	0.94	0.3684	ND	ND

A1 indicates Minor Allele; β, Beta Coefficient; *BCL11A*, B-cell lymphoma/leukemia 11A; BP, Physical Position; ID, Identification; MAF, Minor Allele Frequency; ND, Not Determined; χ2; Chi-square.

Table S2. Fetal hemoglobin association results for 36 SNPs at the HBS1L-MYB locus in Kuwaiti patients with SCD

Locus	SNP ID	Location	MAF	A1	HbF-1		HbF-2		HbF-3		β	P
					χ^2	*P*	χ^2	*P*	χ^2	*P*		
HBS1L-MYB	rs9494145	135111414	0.1287	C	4.23	0.0781	6.33	0.0119	8.487	0.0037	0.71	0.0087
	rs9483788	135114363	0.1435	C	3.96	0.0596	5.14	0.02343	7.964	0.0048	0.63	0.0140
	rs6920211	135110180	0.211	C	2.33	0.2249	3.96	0.04667	7.7	0.0047	0.66	0.0058
	rs4895441	135105435	0.116	G	3.16	0.2300	4.7	0.0300	6.72	0.0090	0.68	0.0173
	rs9389269	135106021	0.116	C	3.16	0.2300	4.70	0.0300	5.90	0.0370	0.64	0.0290
	rs9402686	135106679	0.116	A	3.16	0.2300	4.70	0.0300	6.719	0.0090	0.68	0.0173
	rs4895440	135105420	0.1941	T	1.38	0.3383	2.41	0.1203	8.00	0.0050	0.6775	0.00706
	rs9402685	135098550	0.1646	C	2.41	0.1217	2.62	0.1056	5.94	0.0148	0.55	0.0248
	rs6930223	135103065	0.4916	G	0.78	0.5973	0.00	0.9814	5.72	0.0174	-0.44	0.0232
	rs9376092	135106006	0.1224	A	2.33	0.1320	2.80	0.0900	6.40	0.0100	0.64	0.0204
	rs9494142	135110502	0.1414	C	3.13	0.0800	3.58	0.05	6.32	0.0100	0.61	0.0195
	rs34778774	135097900	0.163	2*	1.949	0.16695	2.4485	0.1185	6.2195	0.01267	0.59	0.02092
	rs11759553	135101158	0.2004	T	0.6323	0.4331	0.8	0.3716	7.236	0.0072	0.65	0.0078
	rs9376090	135090090	0.1097	C	4.20	0.0529	5.47	0.0194	7.08	0.0090	0.70	0.0168
	rs28384513	135055071	0.2068	G	1.5972	0.2728	2.59	0.1074	0.59	0.5138	0.17	0.5089
	rs9389266	135090599	0.1646	T	3.36	0.0693	3.80	0.0513	0.17	0.74	-0.09	0.7348
	rs41294856	135091244	0.1435	T	0.11	0.7478	0.14	0.7043	0.13	0.7832	0.07	0.7872
	rs41294858	135091498	0.1414	C	0.09	0.7580	0.10	0.7557	0.07	0.8015	0.02	0.8057
	rs41294860	135092931	0.1435	G	0.11	0.7478	0.14	0.7043	0.13	0.7832	0.07	0.7871
	rs56076748	135093071	0.1435	A	0.11	0.7478	0.14	0.7043	0.13	0.7832	0.07	0.7872
	rs9494139	135093655	0.1414	G	2.36	0.1409	1.62	0.2037	0.27	0.6717	-0.13	0.6683
	rs55731938	135093712	0.1477	A	0.36	0.6314	0.68	0.4096	0.88	0.3575	0.24	0.3653
	rs7775698	135097497	0.03165	T	0.56	0.5725	1.07	0.3001	0.36	0.5515	-0.37	0.5468
	rs7776196	135097850	0.03165	G	0.10	0.7646	0.17	0.6844	0.16	0.7477	-0.21	0.7447
	rs9402684	135098167	0.4831	T	0.6817	0.5225	0.07	0.7894	3.00	0.0843	-0.32	0.0980
	rs7743042	135098696	0.4789	A	0.6623	0.4880	0.15	0.6971	3.23	0.0725	-0.34	0.0828
	rs1074849	135102274	0.2342	A	0.814	0.4874	0.09	0.7593	0.27	0.7235	0.08	0.7317

	rs1320959	135116248	0.3713	C	2.6135	0.1083	2.30	0.1297	0.11	0.7518	0.06	0.7587
	rs6929404	135132889	0.3397	A	3.693	0.0665	2.65	0.1035	0.46	0.5601	-0.12	0.5723
	rs1022506	135190449	0.0654	A	0.4263	0.5644	0.74	0.3889	0.41	0.5586	0.22	0.5720
	rs6912979	159699767	0.3059	C	0.8902	0.5511	0.01	0.9188	0.54	0.4646	-0.17	0.4504
	rs210796	135189970	0.3523	T	0.2357	0.6355	0.15	0.7027	3.60	0.0612	0.37	0.0689

* rs34778774 genotypes codes (1: CCC, 2: CC)
A1 indicates Minor Allele; β, Beta Coefficient; BP, Physical Position; *HBS1L-MYB*, intergenic region between GTP-binding elongation factor *HBS1L* and myeloblastosis oncogene *MYB*; ID, Identification; MAF, Minor Allele Frequency; ND, Not Determined; SNP: Single Nucleotide Polymorphism; $\chi 2$; Chi-square.

Table S3. Fetal hemoglobin association results for 52 SNPs at the HBB locus in Kuwaiti patients with SCD

Locus	SNP ID	Location	MAF	A1	HbF-1		HbF-2		HbF-3		β	*P*
					χ^2	*P*	χ^2	*P*	χ^2	*P*		
HBB	rs2855039	5250441	0.2131	C	18.13	2.60×10^{-5}	18.22	2.44×10^{-5}	3.39	0.1984	-1.08	0.0002
	rs2071348	5242916	0.2152	T	17.61	3.34×10^{-5}	17.47	3.76×10^{-5}	3.11	0.1849	-1.07	0.0003
	rs2855121	5256061	0.2215	C	16.10	6.65×10^{-5}	15.32	0.000128	3.12	0.2010	-0.99	0.0005
	rs2855122	5256006	0.1983	T	16.64	4.70×10^{-5}	16.33	5.86×10^{-5}	3.74	0.0819	-1.09	0.0003
	rs4910740	5266060	0.1139	A	4.02	0.0453	4.00	0.0458	1.34	0.2538	-0.77	0.0457
	rs7937649	5201149	0.1139	G	13.49	0.0003	11.19	0.0024	1.29	0.4144	-0.93	0.0027
	rs416586	5472327	0.1034	G	13.31	0.0054	7.60	0.0070	2.70	0.1225	-1.15	0.0084
	rs4910743	5288920	0.1772	T	7.35	0.0193	2.58	0.2012	2.41	0.4881	-0.57	0.0491
	rs4601817	5291171	0.1688	A	6.61	0.0197	2.76	0.1604	1.95	0.5163	-0.58	0.0462
	rs3834466	5270333	0.2046	1*	9.17	0.0025	1.82	0.2482	1.16	0.4036	-0.75	0.0180
	rs840716	9432232	0.1308	T	8.11	0.0052	6.97	0.0083	1.06	0.3456	-0.79	0.0130
	rs10837540	5193183	0.1203	T	4.24	0.0481	1.59	0.4924	0.23	0.7020	-0.76	0.0511
	rs549964658	5234551	0.2806	G	8.74	0.0037	6.11	0.0190	0.62	0.4313	-0.74	0.0078
	rs80138317	5234670	0	0	ND	ND	ND	ND	ND	ND	ND	ND
	rs3813727	5234682	0.1013	G	0.32	0.5811	0.29	0.5926	0.01	0.9203	-0.26	0.5692
	rs79582489	5234734	0	0	ND	ND	ND	ND	ND	ND	ND	ND
	rs10837643	5236808	0.1287	A	3.71	0.0578	2.88	0.0906	0.74	0.5022	-0.68	0.0634
	rs4320977	5236932	0.1203	G	4.90	0.0277	4.52	0.0335	1.05	0.3646	-0.85	0.0274
	rs4402323	5237362	0.1224	T	4.58	0.0336	4.07	0.0438	0.88	0.3726	-0.83	0.0327
	rs4910736	5237759	0.1224	A	4.58	0.0336	4.07	0.0438	0.88	0.3726	-0.83	0.0327
	rs968857	5239228	0.1203	C	4.90	0.0277	4.52	0.0335	1.05	0.3646	-0.85	0.0274
	rs968856	5239346	0.1181	C	5.24	0.0225	5.01	0.0252	1.48	0.2636	-0.88	0.0229
	rs10128555	5242347	0.004219	C	0.38	0.5613	0.95	0.3487	4.26	0.0544	ND	ND
	rs1035498826	5242597	0	0	ND	ND	ND	ND	ND	ND	ND	ND
	rs61893081	5242607	0.008439	G	1.06	0.3680	1.94	0.1648	0.94	0.3684	ND	ND
	rs16912210	5242623	0.01055	G	0.95	0.3673	2.39	0.1480	0.61	0.4368	ND	ND

HBB	rs56205611	5248393	0	0	ND	ND	ND	ND	ND	ND	ND	ND
	rs28440105	5248569	0.02321	A	1.74	0.4787	1.81	0.3770	2.75	0.1994	-0.36	0.5494
	rs28379094	5248576	0.1203	T	10.51	0.0013	9.42	0.0022	2.56	0.1342	-1.03	0.0031
	rs2187608	5248701	0.1519	G	14.19	0.0002	11.98	0.0007	1.27	0.3633	-0.98	0.0011
	rs368698783	5249833	0.4662	T	1.27	0.2680	0.71	0.4101	0.05	0.8658	1.63	0.0057
	rs558015287	5249873	0	0	ND	ND	ND	ND	ND	ND	ND	ND
	rs59495893	5252896	0.08439	A	12.20	0.0018	13.81	0.0003	4.05	0.1021	-0.92	0.0100
	rs2070972	5253487	0.1835	C	8.87	0.0047	11.59	0.0007	3.40	0.1632	-1.23	0.0030
	rs11036475	5254010	0.1034	A	2.33	0.1588	4.39	0.0438	1.77	0.2202	-0.62	0.1504
	rs10128653	5256231	0.006329	C	0.57	0.4796	1.43	0.2550	0.44	0.5742	ND	ND
	rs3759070	5270398	0.1034	G	1.20	0.3518	3.00	0.0996	4.38	0.0364	0.14	0.3855
	rs4910742	5285279	0.01899	G	2.54	0.1108	1.33	0.4379	0.24	0.6216	-0.60	0.2712
	rs3886223	5322517	0.08017	T	4.70	0.0407	3.77	0.0540	0.52	0.5175	-0.75	0.0617
	rs4910755	5351521	0.3734	C	0.67	0.5057	0.49	0.5492	0.34	0.6498	0.15	0.5342
	rs4910756	5351626	0.3354	G	0.54	0.5307	1.01	0.4520	0.96	0.3288	0.12	0.5673
	rs7483122	5351776	0.3713	C	0.66	0.4922	0.58	0.5104	0.14	0.7924	0.14	0.5231
	rs5006884	5352021	0.3713	T	0.66	0.4922	0.58	0.5104	0.17	0.7068	0.14	0.5231
	rs5006883	5352081	0.346	C	0.77	0.4266	1.09	0.3800	0.48	0.4891	0.13	0.4632
	rs5024042	5352332	0.3692	A	0.56	0.6319	0.24	0.7301	0.07	0.7920	0.17	0.6519
	rs10500635	5365438	0.3713	A	1.05	0.5170	0.34	0.6494	0.06	0.8622	0.22	0.5404
	rs1391619	5434699	0.1709	G	4.02	0.1347	7.77	0.0062	3.44	0.1317	-0.42	0.1798
	rs10128558	16326884	0.07806	G	0.19	0.7264	0.01	0.9099	1.66	0.2158	0.19	0.7610
	rs3841756	5248304	0.04008	2**	0.11	0.7703	0.26	0.6861	0.15	0.7645	0.28	0.7672
	rs59215941	5242575	0	0	ND	ND	ND	ND	ND	ND	ND	ND
	rs757054403	5248353	0.02186	G	0.08	0.8326	0.42	0.5859	2.55	0.2375	ND	0.4588

* rs3834466 genotypes codes are (1: T, 2: dupT [TT]), ** rs3841756 (1: T, 2: delT)

A1 indicates Minor Allele; β, Beta Coefficient; BP, Physical Position; *HBB*: Beta globin; ID, Identification; MAF, Minor Allele Frequency; ND, Not Determined; SNP: Single Nucleotide Polymorphism; χ2; Chi-square.

Table S4. Fetal hemoglobin association results for 4 SNPs at the ChrX in Kuwaiti patients with SCD

Locus	SNP ID	BP	MAF	A1	HbF-1		HbF-2		HbF-3		β	*P*
					χ^2	*P*	χ^2	*P*	χ^2	*P*		
ChrX	rs6530488	12182662	0.1965	C	1.05	0.3064	0.01	0.9270	0.08	0.7782	-0.25	0.3603
	rs12559632	22142875	0.2971	A	0.24	0.6000	4.91	0.0267	7.86	0.0050	-0.74	0.0080
	rs16981804	22161452	0.0381	T	2.03	0.1540	1.49	0.2221	4.56	0.0328	0.86	0.2062
	rs4969549	24247832	0.2743	G	0.00	0.9548	1.73	0.1886	9.60	0.0080	1.05	0.0030

A1 indicates Minor Allele; β, Beta Coefficient; BP, Physical Position; ChrX: Chromosome X; ID, Identification; MAF, Minor Allele Frequency; ND, Not Determined; SNP: Single Nucleotide Polymorphism; χ2; Chi-square.
